# Virtual BUILD Research Collaboratory: A biomedical data science training using innovative pedagogy to address structures of racism and inequitable stress for undergraduates of color

**DOI:** 10.1371/journal.pone.0294307

**Published:** 2024-02-27

**Authors:** Niquo Ceberio, Peter Le, Jasmón Bailey, Sonthonax Vernard, Nichole Coleman, Yazmin P. Carrasco, Telisa King, Kirsten Bibbins-Domingo, Tung Nguyen, Audrey Parangan-Smith, Kelechi Uwaezuoke, Robert C. Rivers, Kenjus Watson, Leticia Márquez-Magaña, Kala M. Mehta

**Affiliations:** 1 Department of Biology, San Francisco State University, San Francisco, California, United States of America; 2 Applied Physics and Material Sciences, Northern Arizona University, Flagstaff, Arizona, United States of America; 3 School of Education, University of California, Davis, Davis, California, United States of America; 4 University of Maryland—College Park, College Park, Maryland, United States of America; 5 Department of Epidemiology and Biostatistics, University of California, San Francisco, San Francisco, California, United States of America; 6 Skoll Foundation, Palo Alto, California, United States of America; 7 Weill Cornell University of Graduate Medical Sciences, New York, New York, United States of America; 8 Department of Medicine, University of California, San Francisco, San Francisco, California, United States of America; 9 Journal of the American Medical Association, Chicago, Illinois, United States of America; 10 Office of Minority Health Research Coordination, National Institute of Diabetes and Digestive and Kidney Diseases, National Institutes of Health, Bethesda, Maryland, United States of America; 11 American University, Washington, DC, United States of America; Vrije Universiteit Brussel, BELGIUM

## Abstract

**Objective:**

The unprecedented events of 2020 required a pivot in scientific training to better prepare the biomedical research workforce to address global pandemics, structural racism, and social inequities that devastate human health individually and erode it collectively. Furthermore, this pivot had to be accomplished in the virtual environment given the nation-wide lockdown.

**Methods:**

These needs and context led to leveraging of the San Francisco Building Infrastructure Leading to Diversity (SF BUILD) theories of change to innovate a Virtual BUILD Research Collaboratory (VBRC). The purpose of VBRC was to train Black, Indigenous, and people of color (BIPOC) students to apply their unique perspectives to biomedical research. These training activities were evaluated using a pre-post survey design that included both validated and new psychosocial scales. A new scale was piloted to measure culturally relevant pedagogy.

**Results:**

VBRC scholars increased science identity on two items: thinking of myself as a scientist (+1point, p = 0.006) and belonging to a community of scientists (+1point, p = 0.069). Overall, scholars perceived stress also decreased over VBRC (-2.35 points, p = 0.02). Post VBRC, scholars had high agency scores (μ = 11.02, M*d* = 12, range = 6–12, σ = 1.62) and cultural humility scores (μ = 22.11, M*d* = 23, range = 12–24, σ = 2.71). No notable race/ethnic differences were found in any measures.

**Conclusions:**

Taken together, our innovative approach to data science training for BIPOC in unprecedented times shows promise for better preparing the workforce critically needed to address the fundamental gaps in knowledge at the intersection of public health, structural racism, and biomedical sciences.

## Introduction

Efforts to upend deep roots of systemic racism have been far-reaching in terms of discipline, number, and strength; however, many argue that this is only a small barrier to the landslide of inequities that continue to burden Black and Brown people in the United States. In this country, structural inequities permeate its history of unequal access to social mobility because of racist ideals and science education is no different. In recognition of these assertions in 2020, scientists across the country participated in the June 10^th^ #ShutDownAcademia, #ShutDownSTEM, and #Strike4BlackLife protests, which called for white academics and BIPOC to take action towards eradicating anti-Black racism in academia and STEM [1]. Social media, editorials, and some peer-reviewed journals were vessels for an outpouring of stories by Black academics and other BIPOC, detailing the blatantly racist experiences that they have endured within academia [2–6]. Their stories have highlighted how systemic racism has manifested in higher education and has been perpetuated by a non-inclusive culture in science. Moreover, the COVID-19 pandemic brought to the forefront realities of health disparities in BIPOC communities. The disproportionately higher mortality rates of Black people have sparked conversations and calls to action within the science community [7–11].

BIPOC scholars show comparable interest in STEM fields, but such disciplines have not effectively retained these scholars [12–15]. Specifically, Black people earn fewer than 8% of master’s degrees and fewer than 5% of PhDs in STEM fields [16, 17]. Science education has looked the same for much of the last century with learning models centered around lectures (as opposed to discussions/dialogues) [18]. Programs focus on the student deficit model, centering their efforts on building skills with a presumption that scholars are deficient [15], thus shifting accountability to scholars without assessing how educational structures may need to change [15, 19–21]. Most STEM education ignores the community cultural wealth that scholars bring to the institution, minimizing their lived experiences and signaling to BIPOC scholars that only specific skills are pertinent to academic success [22, 23].

There have been some efforts to increase racial and ethnic representation in undergraduate science education through an institutional change (versus a student deficit) lens [24–27]. These include the National Institutes of Health Building Infrastructure Leading to Diversity (NIH-BUILD) which brings together ten universities from across the United States with the overarching goal of increasing entry of undergraduate scholars historically underrepresented in biomedical fields into relevant programs [28]. SF BUILD is one of the ten national NIH-BUILD sites [15, 29–31]. The Virtual BUILD Research Collaboratory (VBRC) was developed out of the SF BUILD program in response to the cancellation of many summer programs in 2020 due to the COVID-19 pandemic [32]. Summer programs are crucial to the success of many scholars, and the cancellation of these programs would have had disproportionate effects on BIPOC scholars. SF BUILD shifted its summer programming to a virtual format and created an online summer research program. Given the disruptions caused by the pandemic, our team had to transform the regular in-person offering into an eight-week online summer coding program, which took place from June 22 –August 13, 2020. The program was then offered to nine sister NIH-BUILD sites. We include a detailed description of the pedagogical components of the VBRC program for leaders in undergraduate education in the US, particularly for those who are attempting to set up summer or short experiences. The objective of the virtual eight-week summer coding program was to teach scholars to use the statistical programming language and software R/RStudio to perform research related to health disparities due to COVID-19 using culturally responsive pedagogy.

To assess the effectiveness of the VBRC program, we evaluated two hypotheses:

Hypothesis 1: Participation in VBRC is associated with increased science identity and intent to pursue bioinformatics.
Hypothesis 2: Participation in VBRC is associated with decreased perceived stress as measured by the perceived stress scale.

## Methods

### Context

The NIH-BUILD initiative consists of ten grants issued to undergraduate institutions to implement and study innovative approaches to engage and retain scholars from diverse backgrounds in biomedical research as a vehicle for them to become future contributors to the NIH-funded research enterprise. NIH-BUILD awards aim to achieve simultaneous impacts at the student, faculty, and institutional levels. Eligibility for NIH-BUILD primary institutions included having fewer than $7.5 million in total NIH research project grant funding and a student population with at least 25 percent Pell Grant recipients. In 2014, 5-year NIH-BUILD awards were issued to 10 undergraduate institutions across the country. In 2019, the second phase of NIH-BUILD began, and the 10 BUILD sites were renewed. SF BUILD, the site in San Francisco, California, was the lead site for VBRC. The site is based on an equal partnership of UCSF, a research-intensive institution, and SFSU, a teaching intensive institution. Having this partnership at the base of an innovative summer program can afford several benefits like high level speakers and detailed evaluation. The Virtual BUILD Research Collaboratory (VBRC) was deemed research exempt from the Institutional Review Board at the University of California, San Francisco (UCSF IRB Approval # 22–37756). Informed consent was not obtained and the UCSF IRB gave permission to use these de-identified educational data for research. No minors took part in the research.

### Culturally responsive pedagogy

VBRC utilized a culturally responsive pedagogical structure that leveraged student’s community cultural wealth [30], and their values to “give back" in science [31–33]. Such linking of social identities to successful performance has been shown in K-12 education and non-STEM disciplines [30]. Preliminarily, in college-level STEM disciplines, the removal of stigma associated with a marginalized social identity increased persistence by signaling ambient belonging in science [30].

### Recruitment & criteria

VBRC was open to undergraduate NIH-BUILD scholars at one of the ten sites nationwide. Recruitment materials explicitly stated that "*No prior coding experience necessary*.” All NIH-BUILD scholars who completed an interest form were invited to participate. Therefore, there was little barrier to entry. The recruitment was open to new NIH-BUILD scholars so at the time of the evaluation the scholars may have had less exposure to other components of the NIH-BUILD interventions.

### Collaborative leadership style

**Fig 1:**
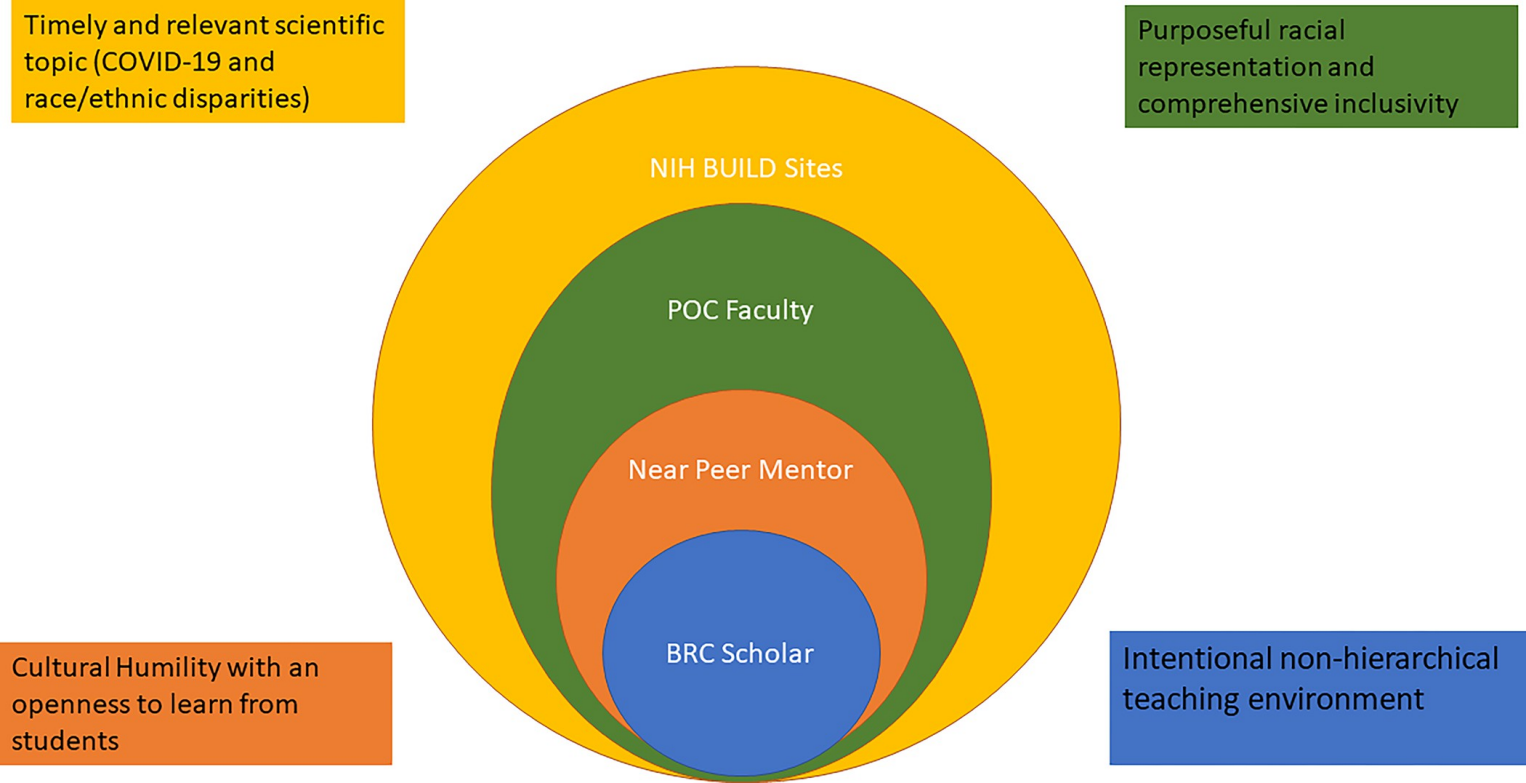
Virtual BUILD Research Collaboratory educational model.

VBRC was developed in collaboration with NIH program officials, SF BUILD staff, near-peer mentors (NPMs), and scholars (Fig 1). The structure of VBRC used a relatively flat hierarchical leadership style, where all participants were encouraged to share ideas, particularly those that would benefit scholars navigating structural or intellectual challenges. Forward-thinking NIH program officials approved the use of SF BUILD unobligated funds to support VBRC for all BUILD scholars because of the shelter-in-place ordinance that meant many would be derailed from their planned summer research experience. This pivot allowed scholars opportunities to meaningfully continue research and professional growth over the summer.

VBRC faculty either came from racial or ethnic groups historically underrepresented in biomedical research or demonstrated a realized commitment towards diversity. The program was held on Zoom from June 22-August 13, 2020 (eight weeks). The program was led Monday-Thursday from 9 am to 1 pm (PST) by one faculty member, three staff members, one NPM coordinator, and eight NPMs. One external faculty member delivered statistics and R coding lectures. The full Student Training Core of the SF BUILD program (three faculty members, two staff members, and two student assistants) met weekly to develop specific inputs for VBRC and to troubleshoot issues. S1 Table describes the engagement speakers and topics included in VBRC.

### Overall structure

VBRC utilized online tools to organize instructional material. All scholars, mentors, and daily staff met each morning in a Zoom "Homeroom’’ where announcements were shared. We purposefully rotated the individuals who shared announcements to affirm a non-hierarchical learning environment. Homeroom also featured music, welcomes, engagement sessions, and statistics/R coding lectures. Approximately two days per week, these sessions were short (~15 minutes), and approximately two days per week, these sessions were longer to deliver educational content (~45 min). After Homeroom, scholars used a separate Zoom link to join their NPM group.

Google Classroom was utilized to hold all course materials and lectures. Though lectures were delivered synchronously, and attendance was highly encouraged, we provided recorded lectures for on-demand review. A Slack platform was utilized for communication outside the two hours of synchronous time. Each NPM group had its own Slack channel that included VBRC leadership, and a general channel allowed for communication between all VBRC participants, faculty, and staff.

Despite the use of multiple tools and practices for regular communication, online learning can be disengaging and not convey that instructors genuinely cared about scholars. To better engage scholars and communicate genuine concern for their professional development and well-being, several activities were implemented. These were varied throughout the program as part of Zoom meetings that addressed emerging issues, which were structured to be stimulating and energetic to simulate an active learning classroom and build camaraderie. Table 1 summarizes these activities.

**Table 1:** Elements of culturally responsive pedagogy in VBRC.

Intervention	When	Elements of Culturally Responsive Pedagogy	Purpose
Opening Ceremony	Daily	Music, word clouds, breakout rooms	Foster community across NPM groups and staff
One-on-One Near Peer Mentor Sessions	Weeks 2–5 of the program	30–40 minutes sessions with NPM (not their lead)	Foster community across NPM groups
A Modern Approach to R and Statistics	Bi-weekly, 30–45 minutes	Real-life examples in lessons	Teach statistical methods in R Studio
Engagement Sessions	Weekly, 45 minutes	Speakers were BIPOC scientists	Signal inclusion and reduce threat

#### Equitable access

Each NIH-BUILD scholar was paired with a NPM and placed in a small group with 5–6 other NIH-BUILD scholars. Groups were randomized with respect to location, so scholars from different sites had an opportunity to build relationships with new people.

The move to online learning can marginalize scholars who may already face disparities in access to technology, a suitable home workspace, etc. With this in mind, we did not require scholars to have their cameras on and encouraged scholars facing technological difficulties to reach out to their mentors or VBRC instructors. We signaled care by taking an active role in troubleshooting hardware issues to ensure access.

On the first day of VBRC, scholars worked with their mentors to install R and RStudio. NPMs contacted scholars who indicated that they did not know if they could run R and RStudio on their computers before starting the program. The NPMs worked with the scholars to find alternatives to run the software. When troubleshooting failed, NPMs worked with members of the VBRC core team to communicate with the scholar’s BUILD site to obtain the necessary equipment. Groups with scholars waiting to receive equipment were accommodated inclusively. For example, one scholar did not have a computer but did have a tablet. In this instance, the scholar, mentor, and VBRC coordinator worked with the student’s BUILD site to order a laptop. While the student was without a computer, the group utilized the Zoom share screen method. Other members of this team took turns writing code while the student without a laptop read coding instructions aloud. The mentor recorded the sessions so the student without the computer could review the code later.

#### No grades

Educational psychologists have systemically studied the effects of grading on students and found that grades tend to (1) diminish student’s interest in whatever they are doing, (2) create a preference for the easiest possible task, (3) and reduce the quality of student’s thinking [34–36]. Therefore, VBRC eliminated the traditional grading structure to affirm that scholars’ interests are held above traditional learning criteria and biased methods of evaluation. This enables scholars to develop and refine their skills through repeated trial and error without consequence.

#### Near Peer Mentors (NPMs)

NPM groups met online for 1.5–2 hours per day, four days a week, for the length of the program. During this time, VBRC scholars interacted with the R and RStudio software to develop research questions and test hypotheses based on their questions. NPMs were selected from the pool of master’s level students engaged in biomedical research at San Francisco State University. Most were from racial and ethnic groups historically underrepresented in biomedical research, and all had recently (within the past 2–4 years) learned to code. NPMs were supported with a toolkit of course materials, a loose learning schedule, and regular access to expert coding assistance.

We encouraged NPMs to collaborate with their teams to develop learning strategies that worked best for their learning styles. The methods used changed depending on the needs and/or desires of the mentees (they were not based on what instructors or mentors felt worked best), and the group’s learning objective. For example, the original curriculum was discarded in favor of tailored worksheets. The original curriculum used the Udacity platform to teach data analysis using the R programming language. Scholars and mentors expressed the need to better present the basics of the coding software in a stepwise fashion to become more confident in its use. Consequently, VBRC instructors and NPMs collaborated to source worksheets that introduced the basics of R/RStudio in a step-by-step approach. Scholars responded positively to the worksheets, providing input for their continued improvement, so the use of worksheets was adopted going forward.

A second example was in the reading materials that were initially assigned to accompany the R statistics course. Many scholars gave feedback to their NPMs that they could not manage these readings alongside their various obligations (some heightened in urgency because of the pandemic) so they were eliminated. Instead, the focus was on engaging with the material in real-time and discussing the application of specific techniques to a particular group’s research question.

NPMs and scholars are given autonomy in their approach to engaging with coding coursework. Some groups preferred a "shut up and code’’ method. Briefly this method starts with scholars sharing aloud what they are going to work on for 25 minutes then they work quietly in breakout rooms of 2–3 people to complete their self-prescribed assignments. If scholars have questions, they leave their breakout rooms and work with their mentor individually in the main Zoom room. Other groups use the share screen method, where all scholars learn the same code as a group with mentor assistance. Regardless of the method, all scholars take a five-minute break at the same time then return to 25 minutes of quiet time. The methods change depending on what the mentees, not faculty or mentors, feel works best.

NPMs, their coordinator, and the program director met via Zoom weekly. These meetings consisted of a debrief to assess the progress of each NPM group. Each NPM would take a turn describing where their mentees were in their coding course, how well their mentees’ needs were being met, and issues their teams were facing. With respect to troubleshooting the issues raised, the weekly meeting allowed for sharing ideas that mentors could try out with their teams. These meetings were also used to identify scholars who were facing technological and attendance issues. This information was used to make real-time changes to VBRC and to take steps to ensure high retention and success in learning to code and conduct community-relevant research.

#### Using community cultural wealth to drive research questions

Scholars were given the overall topic of COVID-19 and health disparities and encouraged to develop research questions relevant to the lived experiences of the communities they come from. Each scholar had a primary research question with a defined predictor, outcome, and testable hypothesis. Scholars had access to a data repository made for the course of all publicly available data on COVID-19, and members of the VBRC team assisted them in mining data for their specific research interests. The near peer mentors also help scholars hone their research questions and guided the work from inception to execution. Consequently, the 8-week summer research program uniquely engaged one of the most pressing societal issue of our time (i.e., COVID-19 pandemic) with the interests and values of scholars to convert anger and/or anxiety to action through data science research.

The first four weeks of the program focused on the fundamentals of coding. The latter half focused on coding particular to the scholar’s research interest. Some groups decided on a cooperative research project, while other teams consisted of one to three scholars. Some scholars collaborated with other groups because of overlapping interests. Therefore, some groups benefited from co-mentoring, with two mentors aiding in their research. At the end of the eight weeks, scholars gave presentations covering their motivation, hypotheses tested, research question, and data source. Furthermore, scholars developed a public health dissemination message to be shared with communities about COVID-19 based on their results.

#### Methods evaluation

VBRC utilized a pre-post survey design delivered via Qualtrics. The survey measured several domains related to the VBRC educational model, including science identity, intention to utilize/pursue bioinformatics/coding in the future, perceived stress immediately after COVID-19 shelter-in-place restrictions and within the past month contextualized by current events; and impact of the different pedagogical structure. S2 Table describes the measures used for VBRC.

#### Science identity

This five-item measure asked how scholars thought about themselves and their science identity and was measured on a 5-point Likert scale (1 = strongly disagree, 5 = strongly agree). A Wilcoxon Signed Ranks test was conducted to assess the pre-and post-median scores for each item.

#### Intent to pursue bioinformatics/coding

This four-item measure specifically probed for scholars’ intent/desire to pursue further bioinformatics/coding opportunities in the future and was measured on a 5-point Likert scale (1 = strongly disagree, 5 = strongly agree). A Wilcoxon was conducted to assess the pre-and post-median scores for each item.

#### Culturally relevant pedagogy

We developed a novel scale to measure two critical constructs of culturally relevant pedagogy. Gloria Ladson-Billings defines culturally relevant pedagogy as one that "empowers students intellectually, socially, emotionally, and politically using cultural referents to impart knowledge, skills, and attitudes” [39]. Ladson-Billings identifies three theoretical constructs of culturally relevant pedagogy: student learning, cultural competence, and critical consciousness. In this way, Black students socially construct their reality through communication and interaction instead of focusing on the margins and borders of the past [37]. Infusing technological advancement into the forward momentum of advancement is consistent with the general empowerment objectives of African American Studies [and its] use of digitized resources in instruction encourage[s] them to think of themselves as potential knowledge producers rather than information consumers [38]. Building on these ideas, a 6-item scale, assessing agency and cultural humility was used. It utilized a 5-point Likert scale; the sub-items are summarized in Table 2 and described further below.

**Table 2:** Culturally relevant pedagogy constructs for the Virtual BUILD Research Collaboratory 2020.

Construct	Item
Cultural Humility	1. I appreciated how the BRC instructors assessed my work. 2. The alternative assessment process (i.e. not relying on traditional graded evaluation or tests) granted me more time to wrestle with the content of the class. 3. I feel supported by the BRC near peer mentors and leadership (staff). 4. The BRC enabled me to investigate racism more rigorously.
Agency	1. I liked developing my own research question. 2. I liked finding “real” data for my research question.

*Agency*. Loosely defined, agency refers to the ability of people, individually and collectively, to exert control over their lives. This is particularly important as it relates to the educational experiences of BIPOC scholars; a sense of agency and ownership in one’s intellectual development is critical towards creating equitable outcomes for BIPOC scholars in STEM. In our evaluation, agency is measured by two statements in our scale with a maximum total score of 12.

*Cultural humility*. In her theoretical model of culturally relevant pedagogy, Ladson-Billings identifies cultural competence as a core tenet defined as "the ability to help students appreciate and celebrate their cultures of origin while gaining knowledge of and fluency in at least one other culture" [39]. However, we maintain that cultural competency is merely not enough to meet the demands of our diverse communities. Much of the dialogue around cultural competency is beginning to shift to the adoption of other cultural frameworks, such as cultural humility. The major limitation posed by the concept of cultural competency is that there is a discrete endpoint to one’s cultural education, which suggests that it can be mastered. However, cultural humility is different in that it is a commitment and active engagement in a lifelong process that individuals enter into on an ongoing basis with patients, communities, colleagues, and with themselves… a process that requires humility in how physicians bring into check the power imbalances that exist in the dynamics of physician-patient communication by using patient-focused interviewing and care" [40]. Thus, we posit that cultural humility is a more appropriate and effective conceptual framework for developing interventions for BIPOC scholars. In our evaluation, cultural humility was measured by four statements on our scale with a maximum total score of 24.

#### Perceived stress scale

Developed by Cohen, et al. [41], the Perceived Stress Scale (PSS) is a widely used and validated tool to measure self-reported psychological stress. We adopted the PSS to assess the effects of the COVID-19 pandemic on perceived stress at two time periods: immediately after shelter-in-place restrictions were implemented (March 2020) and in the past month (August 2020). For our evaluation, PSS was measured on a 7-point Likert scale with a maximum total score of 50.

### Data analysis

We present descriptive characteristics of VBRC scholars and NPMs using chi-square, t-tests, and Mann-Whitney U tests where appropriate. Thereafter, we examine elements of culturally relevant pedagogy—agency and cultural humility—at the conclusion of VBRC. Next, we examine the extent to which race/ethnicity was associated with perceived stress. Lastly, we examine whether the culturally responsive pedagogical structure was associated with decreased stress and/or the impact of critical race theory in education over the course of VBRC.

Because whites were underrepresented in VBRC, we used Latinx/Mexican American/Chican@ as the reference group for our regression analyses. This approach was also utilized to challenge the standardized practice of defining whites (often incorrectly referred to as Caucasians) as the reference group in research [42]. This represents one step towards deconstructing systems of racism built into data analysis.

## Results

### Demographic characteristics

A total of 67 scholars participated in VBRC. Demographic characteristics for scholars (58 scholars and 9 NPMs) are described in Table 3. Many respondents identified as Latinx/Mexican American/Chican@ (46%) or Black/African American (34%); thus, they are overrepresented compared to the general population. S3 Table describes the institutional level participation in VBRC.

**Table 3:** Demographic characteristics of the Virtual BUILD Research Collaboratory 2020.

	Total	Scholars	Near Peer Mentors	p-value
	N = 67	N = 58	N = 9	
BUILD Year				
Incoming new BUILD Scholar	55% (37)	48% (28)	0% (0)	
Returning 2nd Year BUILD Scholar	34% (23)	40% (23)	0% (0)	
Missing	0% (0)	0% (0)	100% (9)	
Other	10% (7)	12% (7)	0% (0)	
**Race and Ethnicity**				0.51
Asian	12% (8)	10% (6)	22% (2)	
Black / African American	34% (23)	38% (22)	11% (1)	
Latinx, Chican@, Mexican American	46% (31)	43% (25)	67% (6)	
Middle Eastern / North African	1% (1)	2% (1)	0% (0)	
Missing	1% (1)	2% (1)	0% (0)	
White	4% (3)	5% (3)	0% (0)	
**Gender**				0.32
Cisgender Female	68% (36)	71% (41)	55% (5)	
Cisgender Male	28% (19)	28% (16)	33% (3)	
Genderqueer/ non-Binary	3% (2)	2% (1)	11% (1)	

### Science identity

Scholars’ sense of science identity increased in nearly all items assessed post VBRC despite relatively high self-reports before participation (Table 3). Based on pre-survey medians for each item, most respondents entered VBRC with a relatively strong sense of science identity (M*d* = 4.00 or 5.00). Scholars’ sense of belonging to a community of scientists increased post VBRC (*Md* = 5.00, *z* = -3.22, p = 0.001, two-tailed) and science identity (M*d* = 5.00, z = -2.77, p = 0.006). Marginally significant findings also indicate an increase in scholars’ sense of belonging in the field of science (M*d* = 5.00, z = -1.82, p = 0.069). There was no change for the item "derive great personal satisfaction from working on a science team that is doing important work" because pre-survey median scores were at the maximum.

### Intent to pursue bioinformatics/coding

Based on the median for each item (4 or 5), most scholars started VBRC with relatively strong intentions to pursue bioinformatics/coding (Table 4). Post-survey medians remained relatively unchanged, with a one-point increase for the item "how likely are you to take more data science classes in the future?" (M*d* = 5.00, z = -3.09, p = 0.002, two-tailed). This may be attributed to the fact that participants entered VBRC with an already existing desire to pursue bioinformatics/coding.

**Table 4:** Median science identity and intent to pursue bioinformatics for the Virtual BUILD Research Collaboratory 2020.

Measure	Pre-test	Post-test	Difference	*z*	*p*
I have a strong sense of belonging to the community of scientists.	4	5	+1	-3.219	0.001*
I derive great personal satisfaction from working on a science team that is doing important work.	5	5	0	-0.715	0.475
I have come to think of myself as a scientist.	4	5	+1	-2.771	0.006*
I feel like I belong in the field of science.	4	5	+1	-1.815	0.069**
The daily work of a scientist is appealing to me.	4	5	+1	-0.989	0.323
Please rate the following from highly unlikely to highly likely—How likely are you to take more data science classes in the future?	4	5	+1	-3.086	0.002*
Please rate the following from highly unlikely to highly likely—How likely are you to pursue graduate training in data science (or a related field)?	4	4	0	-1.992	0.046*
Please rate the following from highly unlikely to highly likely—How likely are you to pursue a career that requires advanced data science or programming skills?	4	4	0	-1.730	0.084**
Please rate the following from highly unlikely to highly likely—How likely are you to contribute substantially to the field of data science (or a related field)?	4	4	0	-1.919	0.055**

*statistically significant

**borderline significant (**scholars only, n = 58).**

### Culturally responsive pedagogy by race/ethnicity

There was a strong sense of agency and cultural humility in VBRC. Descriptive statistics reveal relatively high agency scores (μ = 11.02, M*d* = 12, range = 6–12, σ = 1.62) and cultural humility scores (μ = 22.11, M*d* = 23, range = 12–24, σ = 2.71). In both measures, there were no respondents with a total score that fell below the 50^th^ percentile. However, we are unable to assess the extent to which agency changed throughout VBRC.

Linear regression analyses revealed no significant differences between race/ethnic groups for either agency or cultural humility. However, respondents identified as multiracial yielded a statistically significant result for agency (p = 0.04), indicating a difference in how they experienced agency in VBRC.

### Culturally responsive pedagogy and perceived stress

**Fig 2:**
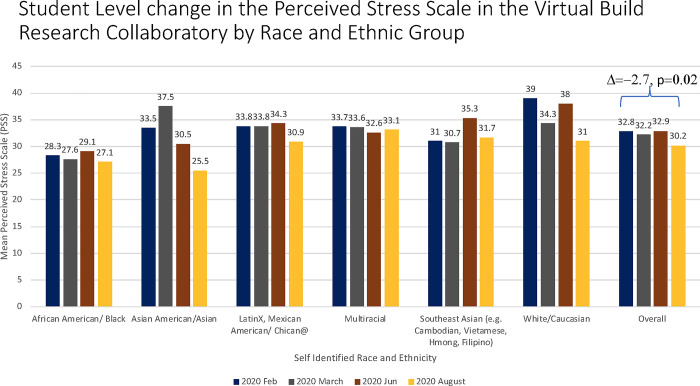
Student level change in the perceived stress scale in the Virtual Build Research Collaboratory by race/ethnicity.

A paired t-test was conducted to determine whether there was a statistically significant mean difference between PSS score at the end of VBRC compared to PSS score at the start of VBRC. Scholars had a slightly lower score at the end of VBRC (30.35 ± 5.88) as opposed to their score at the start of VBRC (32.71 ± 7.15); a statistically significant decrease of -2.35 (95% CI, -4.36 to -0.35), *t*(47) = -2.37, *p* = 0.02. Linear regression analyses revealed no significant differences in change in PSS score between race/ethnic groups (African American / Black: Δ = -2.0, p = 0.58; Asian American / Asian: Δ = -5.0, p = 0.72; Multiracial: Δ = 0.5, p = 0.22; Southeast Asian: Δ = -3.6, p = 0.91; Caucasian: Δ = -7.0, p = 0.32; Overall: Δ = -2.7, p = 0.02) (Fig 2).

Pearson correlation analyses revealed a negative correlation between culturally relevant pedagogy and change in PSS score (difference between PSS score in August 2020 and PSS score in March 2020). That is, as the combined agency and cultural humility score increased, PSS score decreased. However, this was not found to be statistically significant for either agency (r = -0.04, p = 0.77) or cultural humility (r = -0.27, p = 0.071).

## Discussion

Overall, our analyses suggest a statistically significant decrease in perceived stress among VBRC scholars. It also indicates that scholars had relatively similar perceived stress before participation in VBRC. Pre-post change in perceived stress was small, but statistically significant and could be attributable to VBRC, secular trends, or other elements of NIH BUILD not measured by this study. This decrease should be interpreted with caution, as contextually this program took place immediately after the height of the Black Lives Matter movement (Summer 2020) and the post was measured when social restrictions had eased by the end of Summer 2020. In addition, scholars in VBRC were also part of the overall NIH program. That said, the majority were new NIH BUILD scholars and may not have had the opportunity to benefit from other NIH BUILD experiences.

Scholars face a myriad of contributors to stress. For example, they also face racial microaggressions that may contribute to reduced retention of scholars in BIPOC communities [24, 25]. For example, Watson examined the biological effects of racial microaggressions on telomere length among Black male undergraduates at the University of California, Los Angeles. Longer telomeres are predictors of better health outcomes, and shorter telomeres are predictors of shorter lifespans and higher risk for chronic disease. The Watson study found that many of the participants anticipated that they would encounter some form of racial discrimination daily. It also revealed that a high proportion (85–100%) of scholars who attributed everyday discrimination in academia to racism had longer telomere lengths compared to participants who internalized racism (< 46%) [26]. This research suggests that efforts on the part of academic institutions to frame scholars as deficient rather than the institutional environments that limit their success not only push them out of science-related fields, but also worsen health. It also points to the need to create interventions that protect BIPOC student health and increase retention [27, 33].

### Science identity and intent to pursue bioinformatics/coding

Because scholars started with high science identity scores, pre- and post-participation changes are minimal. This may be attributed to VBRC participants entering NIH-BUILD with a strong sense of science identity or having developed this due to participation in NIH-BUILD activities prior to the VBRC summer research experience.

Similarly, the intent to pursue bioinformatics/coding was high at the beginning and end of VBRC. Based on pre-survey medians for each item, most respondents entered VBRC with a strong intent to pursue bioinformatics/coding in the future (M*d* = 4.00), which is not unexpected given that participants opted into the research experience, choosing it over other available training activities. The fact that interest was maintained shows that VBRC was effective in stimulating, sustaining, and catalyzing interest in the field of data science. This result is significant given that traditional STEM education, in the absence of training interventions, has been shown to lead to an overall decrease in intent to pursue advanced study by BIPOC [43].

### Culturally relevant pedagogy

As a novel and non-validated scale, it was challenging to ascertain the impact of culturally relevant pedagogy on scholars, especially given the small sample size. We piloted measures of cultural humility and agency that we hypothesized were expected outcomes of exposure to culturally relevant pedagogy. Using these measures we found high endorsement of both scales, but somewhat unexpected associations between agency and perceived stress as elaborated below. To better understand the relationship between agency and stress, it is important to consider the possibility that while agency is needed to remain in a domain (i.e., field of study) it may also prove detrimental if scholars experience stereotype threat (S.T.) due to the more significant role they have in a domain in which they are stereotyped as incompetent. In other words, given that S.T. is triggered by high-stakes situations where individuals are of high ability and have increased interest in the outcome, it may be that greater agency triggers S.T. for people from stigmatized groups [44]. Consequently, for future evaluation efforts, we intend to expand our scale to include the two-item 7-point Likert measures for S.T. developed by Steele and Aronson: (1) People make judgments about my abilities based on my race, and (2) People make judgments about my racial group based on my performance [45].

### Culturally relevant pedagogy and perceived stress

Our correlation analyses revealed a slight negative association between culturally relevant pedagogy and change in perceived stress score, albeit insignificant. This is consistent with our hypothesis that an increase in agency and cultural humility is correlated with a decrease in change in perceived stress. Similar results between S.T. and racial profiling were found, in that S.T. is reduced when discussing racial profiling in the context of shared learning [46]. In this context, cultural humility appears to operationalize VBRC efforts to examine structural racism (i.e., COVID disparities) in an environment that is non-judgmental, reflective, and about shared learning.

### Change in perceived stress

The decrease in mean difference of perceived stress scale scores (difference = -2.35, p = 0.02) may be attributed to the fact that the perceived stress scale items asked scholars to base their response on current events (i.e., immediately after shelter-in-place and within the past month), which may have been independent of each other. That is, circumstances in March 2020 may have caused a different level of perceived stress versus events in August 2020. Our analyses did not assess for potential correlates of perceived stress at each time point, which may have impacted our findings.

### Limitations

An apparent limitation of this evaluation lies in the sample size of respondents (n = 49), which affords us low statistical power. As a result, some findings did not yield statistical significance and many were marginally significant, demonstrating that despite the unprecedented events of 2020, VBRC scholars made gains. Although this evaluation did not find a significant relationship between the change in perceived stress score and culturally relevant pedagogy, marginally significant findings suggest a relationship between these variables that warrant further investigation.

We were also unable to evaluate the overall change in how scholars experienced the pedagogical structure, as the culturally relevant pedagogy scale was only included in the post-survey. Future evaluation efforts should utilize a pre/post design to assess the extent to which the pedagogical structure influenced agency and cultural humility.

This was a highly specialized program aimed at doing research with scholars, not on scholars. Designed as a summer program with the intent of serving BIPOC scholars in STEM, VBRC did not adopt a traditional experimental design with defined control and experimental groups. However, future iterations of VBRC could be modified to compare NIH-BUILD scholars who participate in the program versus those that do not. We also did not have the opportunity to measure and compare whether VBRC scholars had exposure to the NIH BUILD intervention, particularly at non-San Francisco sites. In San Francisco, all scholars were new BUILD scholars. Additionally, there was no possibility of randomization, as VBRC was an opt-in program open to all NIH-BUILD scholars.

Nevertheless, we successfully enrolled a diverse sample of BIPOC scholars and documented a significant decrease in stress following participation in the VBRC 8-week summer research program. The finding of decreased stress was particularly encouraging as it occurred within the context of the many disruptions and stress due to COVID-19 during the summer of 2020 and highlights the importance of summer programming for BIPOC scholars.

## Conclusion

These evaluation findings contribute to the growing body of literature that inclusive programs are critical and effective in advancing racial equity towards dismantling structures of racism in STEM [47–50]. Based on this work, we recommend that future STEM education programs use and test intentional design with culturally relevant pedagogy aimed at improving agency and cultural humility. Our team plans to build on our novel scale and include other constructs of culturally relevant pedagogy, such as critical consciousness. Other areas of study include evaluating other variables, such as the number of years of involvement in NIH-BUILD.

## Supporting information

S1 Table Engagement speakers and topics for the virtual BUILD Research Collaboratory 2020. (DOCX)

S2 Table Evaluation measures for the Virtual BUILD Research Collaboratory 2020. (DOCX)

S3 Table Institutional level participation in the BUILD Research Collaboratory (n = 65). (DOCX)
