# Supplementary material for: Virtual BUILD Research Collaboratory: A biomedical data science training using innovative pedagogy to address structures of racism and inequitable stress for undergraduates of color
Source: PLoS One. 2024 Feb 27;19(2):e0294307. doi: 10.1371/journal.pone.0294307 (PMC10898773; doi:10.1371/journal.pone.0294307)
Supplement: S1 Table — (DOCX) [file pone.0294307.s001.docx]

**Supporting Information**

**S1 Table. Engagement Speakers and Topics for the Virtual BUILD Research Collaboratory 2020.**

| **Engagement Speaker** | **Topic** |
| --- | --- |
| Dr. Kirsten Bibbins-Domingo | Epidemiology and Public Health |
| Dr. Kenjus Watson and Nina Monet | Critical Race Theory |
| Dr. Alicia Fernandez | COVID-19 and its impact on our most vulnerable populations |
| Dr. Leticia Márquez-Magaña | Converting Microaggressions into Microaffirmations |
| Dr. George Rutherford | COVID-19 Statistics Nationwide |
| Dr. Tung T. Nguyen | Asian Americans and COVID-19 |
| Dr. Alain Bonny | COVID-19 Immune Response |
| Dr. Suzanna Martinez | COVID-19 on College Campuses |
| Dr. Andi Egbert and Gilbert Cortez  Dr. Kelechi Uwaezuoke  Dr. Audrey Parangan-Smith  Dr. Peter Chin-Hong  Miguel Márquez, M.P.P., J.D. | A.P.M. Research Color of COVID-19 Data  Community Health Engagement Tool-Kit  Critical Digital Literacy  Frontline on COVID-19  Santa Clara County’s Response to COVID-19 |
