# Supplementary material for: Virtual BUILD Research Collaboratory: A biomedical data science training using innovative pedagogy to address structures of racism and inequitable stress for undergraduates of color
Source: PLoS One. 2024 Feb 27;19(2):e0294307. doi: 10.1371/journal.pone.0294307 (PMC10898773; doi:10.1371/journal.pone.0294307)
Supplement: S2 Table — (DOCX) [file pone.0294307.s002.docx]

**S2 Table. Evaluation Measures for the Virtual BUILD Research Collaboratory 2020.**

**Collaboratory 2020.**

| **Measure** | **# of items** | **Likert scale** | **Range of scores** |
| --- | --- | --- | --- |
| Science identity | 5 | 5-point (1-5) | 5 – 25 |
| Intention to utilize/pursue bioinformatics/coding in the future | 4 | 5-point (1-5) | 5 – 20 |
| Perceived stress immediately after COVID-19 shelter-in-place restrictions | 10 | 5-point (1-5) | 5 – 50 |
| Perceived stress in the past month with current events | 10 | 5-point (1-5) | 5 – 50 |
| Impact of the culturally responsive pedagogical structure  (post-survey only) | Agency (2)  Cultural Humility (4) | 7-point (0-6) | Agency: 0 – 12  Cultural Humility: 0 – 24 |
