# Supplementary material for: Virtual BUILD Research Collaboratory: A biomedical data science training using innovative pedagogy to address structures of racism and inequitable stress for undergraduates of color
Source: PLoS One. 2024 Feb 27;19(2):e0294307. doi: 10.1371/journal.pone.0294307 (PMC10898773; doi:10.1371/journal.pone.0294307)
Supplement: S3 Table — (DOCX) [file pone.0294307.s003.docx]

| **S3. Institutional Level participation in the BUILD Research Collaboratory (n=65)** | | | |
| --- | --- | --- | --- |
| **Institutions (7/10)** | **Participated in VBRC** | **Number of Scholars** | **Percentage** |
| San Francisco State University: [SF BUILD](http://sfbuild.sfsu.edu/): Enabling Scholars to Represent in Science | **Yes** | **21** | **32.3** |
| California State University, Long Beach: [CSULB BUILD](http://www.csulb.edu/build) | **Yes** | **3** | **4.6** |
| California State University, Northridge: [BUILD PODER](http://www.csun.edu/build-poder) (Promoting Opportunities for Diversity and Education and Research) | **Yes** | **3** | **4.6** |
| Morgan State University: [BUILD ASCEND](http://www.morgan.edu/ASCEND) (A Student-Centered, Entrepreneurship Development Training Model to Increase Diversity in the Biomedical Research Workforce) | **Yes** | **2** | **3.1** |
| Portland State University: [BUILD EXITO](http://www.pdx.edu/exito/) (Enhancing Cross-Disciplinary Infrastructure Training at Oregon) | **Yes** | **6** | **9.2** |
| University of Detroit, Mercy: [ReBUILD Detroit](http://rebuildetroit.org/) | **Yes** | **23** | **35.4** |
| Wayne State University | **Yes** | **4** | **6.2** |
| The University of Texas at El Paso: [BUILDing SCHOLARS](http://buildingscholars.utep.edu/web/) (Southwest Consortium of Health-Oriented Education Leaders and Research Scholars) | **Yes** | **3** | **4.6** |
| University of Alaska, Fairbanks: [BUILD BLaST](https://alaska.edu/blast/) (Biomedical Learning and Student Training) | **No** |  |  |
| University of Maryland, Baltimore County: [STEM BUILD at UMBC](http://stembuild.umbc.edu/) | **No** |  |  |
| Xavier University of Louisiana: [Project Pathways​](https://www.xula.edu/researchbuild) | **No** |  |  |

Legend. [SF BUILD](http://sfbuild.sfsu.edu/): Enabling Scholars to Represent in Science, SFSU San Francisco State University; [CSULB BUILD](http://www.csulb.edu/build): California State University, Long Beach; [BUILD PODER](http://www.csun.edu/build-poder) (Promoting Opportunities for Diversity and Education and Research) California State University, Northridge; [BUILD ASCEND](http://www.morgan.edu/ASCEND): Morgan State University: (A Student-Centered, Entrepreneurship Development Training Model to Increase Diversity in the Biomedical Research Workforce); [BUILD EXITO](http://www.pdx.edu/exito/): Enhancing Cross-Disciplinary Infrastructure Training at Oregon, Portland State University; [ReBUILD Detroit](http://rebuildetroit.org/), University of Detroit, Mercy; Wayne: Wayne State University; UTEP: the University of Texas at El Paso: [BUILDing SCHOLARS](http://buildingscholars.utep.edu/web/) (Southwest Consortium of Health-Oriented Education Leaders and Research Scholars); UCSF: the University of California, San Francisco; A.P.M.: American Public Media
